# Effects of COVID-19 on Maternal and Neonatal Outcomes and Access to Antenatal and Postnatal Care, Malawi

**DOI:** 10.3201/eid2910.230003

**Published:** 2023-10

**Authors:** Leonard Mndala, Chikondi Chapuma, Jennifer Riches, Luis Gadama, Fannie Kachale, Rosemary Bilesi, Malangizo Mbewe, Andrew Likaka, Moses Kumwenda, Regina Makuluni, Bertha Maseko, Chifundo Ndamala, Annie Kuyere, Laura Munthali, Deborah Phiri, Edward J.M. Monk, Marc Y.R. Henrion, Maria L. Odland, David Lissauer

**Affiliations:** Malawi-Liverpool-Wellcome Programme, Blantyre, Malawi (L. Mndala, C. Chapuma, J. Riches, M. Kumwenda, R. Makuluni, B. Maseko, C. Ndamala, A. Kuyere, L. Munthali, D. Phiri, E.J.M. Monk, M.Y.R. Henrion, M.L. Odland, D. Lissauer);; University of Liverpool, Liverpool, UK (L. Mndala, C. Chapuma, J. Riches, M.L. Odland, D. Lissauer);; Kamuzu University of Health Sciences, Blantyre (L. Gadama, M. Kumwenda);; Ministry of Health, Lilongwe, Malawi (F. Kachale, R. Bilesi, M. Mbewe, A. Likaka);; Universidade de Pernambuco, Recife, Brazil (A. Likaka);; Liverpool School of Tropical Medicine, Liverpool (M.Y.R. Henrion);; St. Olavs University Hospital, Trondheim, Norway (M.L. Odland)

**Keywords:** COVID-19, respiratory infections, severe acute respiratory syndrome coronavirus 2, SARS-CoV-2, SARS, coronavirus disease, zoonoses, viruses, coronavirus, maternal health, Surveillance, interrupted time series, Malawi, Africa

## Abstract

We used national facility-level data from all government hospitals in Malawi to examine the effects of the second and third COVID-19 waves on maternal and neonatal outcomes and access to care during September 6, 2020–October 31, 2021. The COVID-19 pandemic affected maternal and neonatal health not only through direct infections but also through disruption of the health system, which could have wider indirect effects on critical maternal and neonatal outcomes. In an interrupted time series analysis, we noted a cumulative 15.4% relative increase (63 more deaths) in maternal deaths than anticipated across the 2 COVID-19 waves. We observed a 41% decrease in postnatal care visits at the onset of the second COVID-19 wave and 0.2% by the third wave, cumulative to 36,809 fewer visits than anticipated. Our findings demonstrate the need for strengthening health systems, particularly in resource-constrained settings, to prepare for future pandemic threats.

Maternal, newborn, and child health delivery services are critical, and many improvements in health outcomes have been achieved globally in the past decade (*1*). However, those gains likely were negatively affected by the COVID-19 pandemic and subsequent public health response. The pandemic disrupted global healthcare systems and healthcare delivery, even in the most well-resourced and resilient health systems (*2*). In the worst-case scenario, COVID-19’s disruption of essential maternal, newborn, and child health interventions could result in an additional 1,157,000 child deaths and 56,700 maternal deaths in 118 low- and middle-income countries (*3*). For instance, in Nepal, the national COVID-19 lockdown led to an increase in stillbirth and neonatal mortality rates and decreases in quality of care in healthcare institutions (*4*).

Most countries in sub-Saharan Africa have struggling healthcare systems (*5*). Pandemics exacerbate existing challenges. For instance, the 2014 Ebola outbreak in West Africa greatly affected maternal and child health services and fewer women attended antenatal care visits, fewer institutional deliveries occurred, and child vaccination coverage was low (*6*). Although risks to achieving improvements in maternal, neonatal, and child health outcomes posed by COVID-19 are well recognized, little national-level evidence has been collected to elucidate the wider effects of COVID-19 waves on maternal, neonatal, and child health outcomes in sub-Saharan Africa (*3*).

In Malawi, a SARS-CoV-2 seroprevalence study of 5,085 blood samples from blood donors showed that seroprevalence was 70.2% (95% CI 62.2%–81.6%) by July 2021 (*7*). The Ministry of Health (MoH) prioritized the COVID-19 response, but because of existing challenges in the healthcare infrastructure, the pandemic greatly disrupted healthcare delivery (*8*). A paucity of data are available from sub-Saharan Africa to establish the effects of COVID-19 on maternal, neonatal, and child health. In response, Malawi MoH collaborated with the Malawi-Liverpool-Wellcome Programme (MLW) to implement the Maternal COVID-19 Surveillance (MATSurvey) online platform to routinely collect and monitor data on COVID-19 in maternity.

Limited research has been conducted in Malawi and sub-Saharan Africa using national real-time data collected during the peak of the pandemic to assess effects of COVID-19 on maternal and neonatal health and access to healthcare. We analyzed the effects of the second and third COVID-19 waves in Malawi on selected maternal and neonatal outcomes and access to care by using nationally collected data from all 33 government healthcare sites participating in MATSurvey. 

## Methods

### Study Design

This study is a secondary retrospective cohort analysis of data gathered from a cohort of pregnant and recently pregnant women enrolled in the MATSurvey platform from September 6, 2020–October 31, 2021. Data from the platform are routinely collected for all women admitted in all 33 government healthcare facilities of Malawi, including all 27 government district hospitals, all 4 central hospitals, and 2 district health offices.

### Data Source

MATSurvey was implemented by using available structures within Malawi’s healthcare system. MATSurvey involved digitization of existing Maternal Death Surveillance and Response (MDSR) program tools and reviews. Digitization enabled electronic data capture in all government facilities during COVID-19, making data available in real-time for monitoring, evaluation, and response. Prior to MATSurvey, data collection under MDSR was paper-based. MATSurvey comprises all facilities under MDSR. The MDSR site coordinators are responsible for daily active case-finding using data from facility teams, clinical notes, hospital registers, and handover files. Cases include women who have died, suffered a maternal near-miss event (i.e., almost died from complications), or had suspected or confirmed COVID-19.

Site coordinators were trained in digital data collection and entry for a tailored electronic tablet application developed by the MLW team. Designated project coordinators provided oversight of the project from MLW remotely, together with MoH’s quality management directorate zonal coordinators, to assist with timeliness in uploading data and addressing challenges. MLW and MoH conducted weekly data quality checks.

### Definitions and Measurements

**Figure 1:**
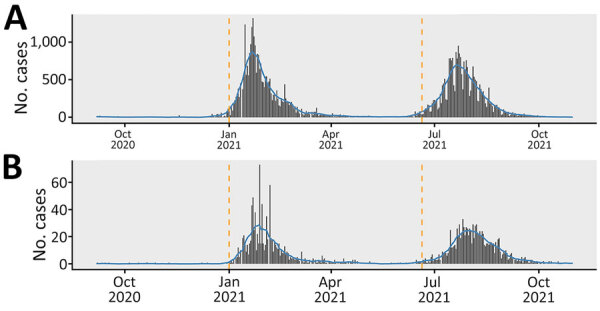
Epidemic data used in study of effects of COVID-19 on maternal and neonatal outcomes and access to antenatal and postnatal care, Malawi. A) Daily confirmed COVID-19 cases; B) daily confirmed COVID-19 deaths. The epidemiologic curve shows the beginning of second and third waves of COVID-19 in Malawi. Grey bars indicate daily case counts; blue lines indicate centered 14-day moving averages; orange vertical lines indicate proposed time points for the interruptions in the segmented time series analysis: January 1, 2021, just before the second COVID-19 wave; and June 20, 2021, just before the third COVID-19 wave. Data are from the Johns Hopkins University Center for Systems Science and Engineering (https://coronavirus.jhu.edu/map.html).

Using the COVID-19 epidemic curve for Malawi (Figure 1), we defined the baseline period as September 6–December 31, 2020; the second wave as January 1–June 19, 2021; and the third wave as June 20–October 31, 2021. We defined maternal death rate (deaths/1,000 live births) as the number of deaths from any cause related to or aggravated by pregnancy or its management, excluding accidental or incidental causes, during pregnancy and childbirth or within 42 days after termination of pregnancy, irrespective of the duration and site (e.g., ectopic) of the pregnancy. We defined neonatal death rate (deaths/1,000 live births) as the number of newborns up to 28 days of age who died before discharge. We defined stillbirth rate (stillbirths/1,000 live births) as the number of fetal deaths occurring during the antepartum and intrapartum period, after 28 weeks of pregnancy. We aggregated the number of antenatal clinic visits per week across the study period. We defined postnatal clinic visits as aggregated counts of visits per week across the study period. We defined the counterfactual scenario as the period during which no effects from second and third COVID-19 waves were seen.

### Statistical Analysis

We aggregated data weekly across all facilities. We limited the date range of our analysis to September 6, 2020, after the end of the first COVID-19 wave in Malawi, to October 31, 2021, before the start of the fourth COVID-19 wave (Figure 1).

To assess the overall effects of the second and third pandemic waves on access to care and maternal and neonatal outcomes, we used an interrupted times series (ITS) regression approach for the time series obtained for each outcome (*9*). We used a negative binomial regression model for weekly reported clinic visits and maternal and neonatal outcomes. Those models included a linear trend over time for the logarithm of the rate of observed events. The ITS framework can foresee that a linear trend can be interrupted at >1 timepoints, both in overall level of events and in trend over time after interruptions. In our study, we allowed 2 interruptions: January 1, 2021, which was the start of second COVID-19 wave in Malawi; and June 20, 2021, the start of third COVID-19 wave. During the first COVID-19 wave in Malawi, MATSurvey was just being rolled out and not fully functional for capturing data. During that phase, data on COVID-19 in maternity were unavailable. Because of the paucity of data, we chose the period just after the first wave as the counterfactual period; that is, the period in which no effects of the second and third COVID-19 waves were seen. At each of the 2 interruption timepoints, we allowed both the level of the modeled events and the slope of the trajectory of number of events over time to change. For outcomes for which we estimated rates per 1,000 live births, the models also included an offset (i.e., a denominator) term for the logarithm of the number of weekly live births. To avoid ambiguity, and for the interest of mathematically inclined readers, we have made the precise model equations and code available (https://github.com/gitMarcH/MatSurv_ITS).

We also fitted models to data restricted to the time range up to the first interruption time point. Those models only included a constant term (the intercept), time, and the live birth denominator as an offset, if applicable. We used those models only for the first of the 3 time windows we considered in our analyses; that is, the period before the first interruption time point. By using those models to predict beyond the first interruption, the second COVID-19 wave, we were able to compare the counterfactual scenario of no effect on outcomes from the 2 COVID-19 waves against the observed events. To quantify the relative increase or decrease between the counterfactual model predictions and observed events, we divided the difference between both by the number of observed events. We characterized all outcomes in this study by a substantial amount of variation not explained by our models, which are most likely because covariates and confounders that are not recorded in the MATSurvey dataset. We quantified uncertainty in estimates by using the 95% CIs for the extrapolated model fits for the mean number of events for each outcome variable.

For model diagnostics, we inspected deviance residuals against fitted values and checked autocorrelations and partial autocorrelations for substantial deviations from model assumptions. We also computed pseudo R^2^ values for every model. We chose January 1, 2021, and June 20, 2021, as timepoints by visually inspecting the epidemic curve (Figure 1). We performed analyses by using R version 4.1.2 (*10*). We fit negative binomial models for the ITS analyses by using the glm.nb() function from the library of Modern Applied Statistics with S (*11*).

### Ethics Statement

Data were anonymized and made available to the research team by permission of MoH, Malawi, and the College of Medicine Research Ethics committee (protocol no. P.11/20/3186). Only aggregate data were made available to maintain patients’ confidentiality. Patient consent was not required because MATSurvey is a national platform owned by MoH for routinely collecting data.

## Results

During September 6, 2020–October 31, 2021, Malawi recorded 589 maternal deaths across all the healthcare facilities registered in MATSurvey. Of those deaths, 176 (29.9%) occurred during the baseline period, 208 (35.3%) occurred during the second COVID-19 wave, and 205 (34.8%) occurred during the third COVID-19 wave. During the same timeframe, Malawi recorded 6,701 neonatal deaths, of which 40.2% (n = 2,695) occurred during the second COVID-19 wave. The country reported 280,246 antenatal and 108,320 postnatal clinic visits. During the same timeframe, Malawi had 226,057 births, of which 67,377 (29.8%) were during the baseline, 88,685 (39.2%) were during the second wave, and 69,995 (31%) were during the third wave.

### Maternal Death Rate

**Figure 2:**
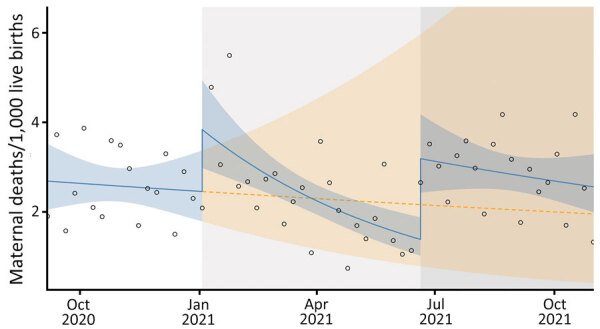
Maternal death rates in study of effects of COVID-19 on maternal and neonatal outcomes and access to antenatal and postnatal care, Malawi. Circles indicate observed data; blue lines indicate model fit from actual data, including step and slope changes during second (January 1, 2021) and third (June 20, 2021) COVID-19 waves. Dashed orange line indicates the counterfactual scenario of no second or third COVID−19 waves. Blue shaded areas indicate 95% CIs; yellow shaded areas indicate 95% CIs for the counterfactual scenario. Background shaded areas indicate the second (light gray) and third (dark gray) COVID−19 waves in Malawi. Pseudo R^2^ = 0.06.

Compared with maternal deaths during September–December 2020 (baseline), a substantial increase occurred during January–October 2021, across the second and third COVID-19 waves (Figure 2). At the beginning of the second COVID-19 wave, we observed an increase in maternal deaths, which remained above the counterfactual scenario until around May 2021. We observed another increase in the outcome at the beginning of third wave, around July 2021.

The second COVID-19 wave led to a 57% increase in maternal death rate (rate ratio [RR] 1.57; p = 0.0228) compared with the counterfactual scenario. Maternal death rate increased 2-fold after the third COVID-19 wave (RR 2.31; p<0.001). The increase in maternal death rate across the second and third COVID-19 waves was accompanied by a sustained post–third wave effect (RR 1.03; p = 0.0408) (Table 1).

**Table 1:** Summary of regression analysis for maternal death rate in study of effects of COVID-19 on maternal and neonatal outcomes and access to antenatal and postnatal care, Malawi*

Variable	Estimate (95% CI)†	p value
Baseline, intercept	2.69 (2.00–3.63)	
COVID-19 wave 2	1.57 (1.06–2.30)	**0.0228**
COVID-19 wave 3	2.31 (1.54–3.47)	**0.0001**
Time, wk	0.99 (0.97–1.03)	0.7296
Post–COVID-19 wave 2	0.96 (0.93–0.99)	**0.0438**
Post–COVID-19 wave 3	1.03 (1.00–1.06)	**0.0408**

*Pseudo R^2^ = 0.06. Bold text indicates statistical significance. †The estimates are exponentiated model coefficients, i.e., the intercept estimate is the baseline incidence rate per 1,000 live births and all other estimates are rate ratios associated with the different variables.

The total difference in maternal death rate between the counterfactual scenario and observed numbers from the beginning of the second through the third wave was −63 (95% CI −264 to 533). That finding represents an absolute increase by 63 maternal deaths and a relative increase of 15.4% during combined second and third waves of COVID-19 compared with the counterfactual scenario.

### Neonatal Death Rate

**Figure 3:**
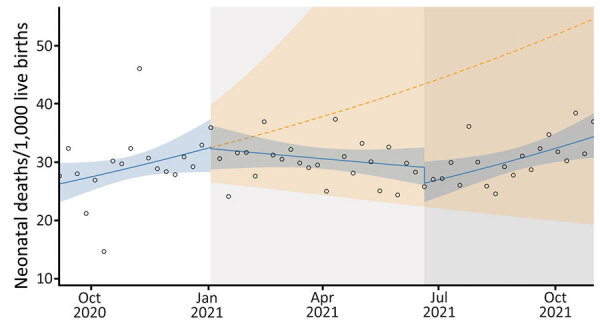
Neonatal death rates in study of effects of COVID-19 on maternal and neonatal outcomes and access to antenatal and postnatal care, Malawi. Circles indicate observed data; blue lines indicate model fit from actual data, including step and slope changes during second (January 1, 2021) and third (June 20, 2021) COVID-19 waves. Dashed orange line indicates the counterfactual scenario of no second or third COVID−19 waves. Blue shaded areas indicate 95% CIs; yellow shaded areas indicate 95% CIs for the counterfactual scenario. Background shaded areas indicate the second (light gray) and third (dark gray) COVID−19 waves in Malawi. Pseudo R^2^ = 0.02.

At the beginning of the second COVID-19 wave, we observed no statistically significant change in the neonatal mortality rate (Figure 3). However, we saw a reduction at the beginning of the third wave. After the second COVID-19 wave, we saw a sustained decrease in the neonatal death rate, but we noted a sustained increase in neonatal death rate after the third wave (Table 2).

**Table 2:** Summary of regression analysis for neonatal death rate in study of effects of COVID-19 on maternal and neonatal outcomes and access to antenatal and postnatal care, Malawi*

Variable	Estimate (95% CI)†	p value
Baseline, intercept	25.93 (22.55–29.82)	
COVID-19 wave 2	0.99 (0.83–1.17)	0.9358
COVID-19 wave 3	0.91 (0.76–1.07)	0.2601
Time, wk	1.01 (0.99–1.02)	0.0691
Post COVID-19 wave 2	0.98 (0.96–0.99)	**0.0361**
Post COVID-19 wave 3	1.02 (1.0–1.03)	**0.0080**

*Pseudo R^2^ = 0.02. Bold text indicates statistical significance. †The estimates are exponentiated model coefficients, i.e., the intercept estimate is the baseline incidence rate per 1,000 live births and all other estimates are rate ratios associated with the different variables.

We observed a statistically nonsignificant drop in neonatal deaths at the interruption of both the second (RR 0.99; p = 0.9358) and third (RR 0.91; p = 0.2601) COVID-19 waves in Malawi. Both waves were associated with a change in time trend: a 2% drop occurred in the weekly trend during the second wave (RR 0.98; p = 0.0361), resulting in a slight decrease over time during that wave, then a 2% increase in the weekly trend, resulting again in a sustained increase in neonatal deaths over time during the period after the third wave (RR 1.02; p = 0.008).

The difference in neonatal mortality rates between the counterfactual scenario and observed outcomes from January 1–October 31, 2021, was 1,988 (95% CI −1,180 to 8,715). That finding translates to an absolute reduction of 1,988 and relative reduction of 41.4% neonatal deaths across the second and third COVID-19 waves.

### Stillbirth Rate

**Figure 4:**
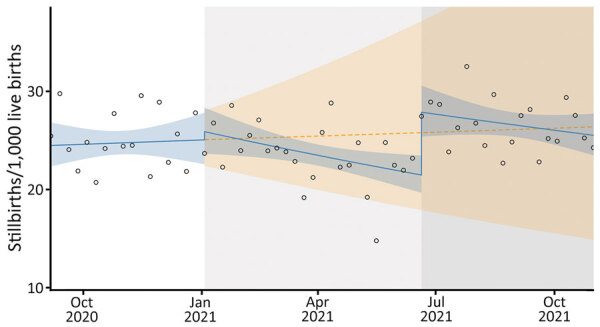
Stillbirth rates in study of effects of COVID-19 on maternal and neonatal outcomes and access to antenatal and postnatal care, Malawi. Circles indicate observed data; blue lines indicate model fit from actual data, including step and slope changes during second (January 1, 2021) and third (June 20, 2021) COVID-19 waves. Dashed orange line indicates the counterfactual scenario of no second or third COVID−19 waves. Blue shaded areas indicate 95% CIs; yellow shaded areas indicate 95% CIs for the counterfactual scenario. Background shaded areas indicate the second (light gray) and third (dark gray) COVID−19 waves in Malawi. Pseudo−R^2^ = 0.04.

We observed 131 (95% CI −1,032 to 1,863) fewer stillbirths per 1,000 live births compared with a scenario of no second and third COVID-19 waves. The drop in stillbirths was more of a sustained effect and not an immediate result of the second and third wave interruptions (Table 3; Figure 4). An immediate 30% increase in stillbirths occurred at the onset of the third COVID-19 wave (RR 1.30; p<0.001), but the increase was not sustained.

**Table 3:** Summary of regression analysis for stillbirths in study of effects of COVID-19 on maternal and neonatal outcomes and access to antenatal and postnatal care, Malawi*

Variable	Estimate (95% CI)†	p value
Baseline, intercept	22.47 (22.14–27.04)	
COVID-19 wave 2	1.04 (0.91–1.18)	0.6225
COVID-19 wave 3	1.30 (1.14–1.47)	**0.0001**
Time, wk	1.00 (0.99–1.01)	0.7905
Post COVID-19 wave 2	0.99 (0.98–1.00)	0.1304
Post COVID-19 wave 3	1.00 (0.99–1.01)	0.5393

*Pseudo R^2^ = 0.04. Bold text indicates statistical significance. †The estimates are exponentiated model coefficients, i.e., the intercept estimate is the baseline incidence rate per 1,000 live births and all other estimates are rate ratios associated with the different variables.

### Antenatal and Postnatal Clinic Visits

**Figure 5:**
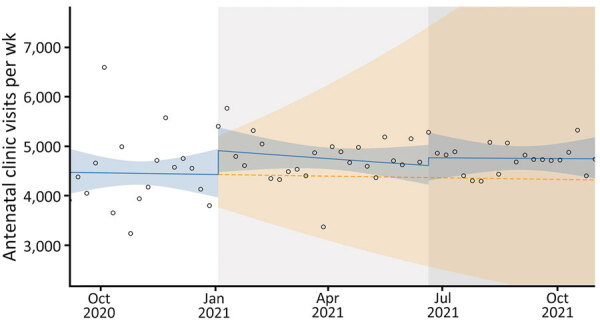
Antenatal clinic visits in study of effects of COVID-19 on maternal and neonatal outcomes and access to antenatal and postnatal care, Malawi. Circles indicate observed data; blue lines indicate model fit from actual data, including step and slope changes during second (January 1, 2021) and third (June 20, 2021) COVID-19 waves. Dashed orange line indicates the counterfactual scenario of no second or third COVID−19 waves. Blue shaded areas indicate 95% CIs; yellow shaded areas indicate 95% CIs for the counterfactual scenario. Background shaded areas indicate the second (light gray) and third (dark gray) COVID−19 waves in Malawi. Pseudo R^2^ = 0.01.

**Figure 6:**
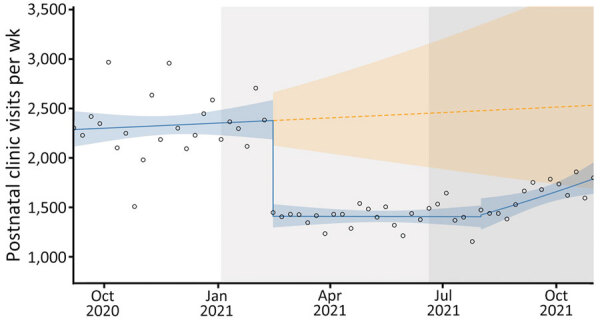
Postnatal clinic visits in study of effects of COVID-19 on maternal and neonatal outcomes and access to antenatal and postnatal care, Malawi. Circles indicate observed data; blue lines indicate model fit from actual data, including step and slope changes during second (January 1, 2021) and third (June 20, 2021) COVID-19 waves. Dashed orange line indicates the counterfactual scenario of no second or third COVID−19 waves. Blue shaded areas indicate 95% CIs; yellow shaded areas indicate 95% CIs for the counterfactual scenario. Background shaded areas indicate the second (light gray) and third (dark gray) COVID−19 waves in Malawi. Pseudo R^2^ = 0.12.

During the beginning of the second and third COVID-19 waves, antenatal clinic visits increased (Figure 5), but we observed a sharp drop in postnatal clinic visits at the onset of the second wave (Figure 6). During the second and third COVID-19 waves, January 1, 2021–October 31, 2021, we observed an increase of 16,833 (95% CI 89,072–111,041) more antenatal clinic visits than during the period without the 2 waves. Although the differences were not statistically significant, during the second COVID-19 wave, we noted an 11% (RR 1.11; p = 0.1395) increase in antenatal clinic visits, but only a 3% (RR 1.03; p = 0.6187) increase occurred during the third COVID-19 wave.

Postnatal clinic visits dropped by 41% at the onset of the second COVID-19 wave (RR 0.59; p<0.001). A 0.2% decrease in postnatal care visits occurred at the onset of the third COVID-19 wave (RR 1.02; p = 0.0149), but although that decrease was not statistically significant, it was accompanied by a sustained post–third wave effect (Table 4). Postnatal clinic visits declined by 36,809 (95% CI 15,799–64,848) visits between the counterfactual scenario and observed outcome.

**Table 4:** Summary of regression analysis for weekly postnatal visit rate in study of effects of COVID-19 on maternal and neonatal outcomes and access to antenatal and postnatal care, Malawi*

Variable	Estimate (95% CI)†	p value
Baseline, intercept	2,283.98 (2,100.42–2,483.58)	
COVID-19 wave 2	0.59 (0.53–0.66)	**0.0000**
COVID-19 wave 3	1.01 (0.88–1.16)	0.8521
Time, wk	1.00 (0.99–1.01)	0.5854
Post COVID-19 wave 2	0.99 (0.98–1.00)	0.6701
Post COVID-19 wave 3	1.02 (1.00–1.03)	**0.0149**

*Pseudo R^2^ = 0.12. Bold text indicates statistical significance. †The estimates are exponentiated model coefficients, i.e., the intercept estimate is the baseline rate, and all other estimates are rate ratios associated with the different variables.

## Discussion

Using an ITS regression analysis of aggregated time series data on COVID-19 in maternity, we estimated the total effects of the second and third COVID-19 waves on selected maternal and neonatal outcomes, and access to antenatal and postnatal care in Malawi. We found a statistically significant increase in the maternal death rate at the beginning of the second and third COVID-19 waves compared with a counterfactual scenario of no second and third COVID-19 waves. However, the neonatal death rate was largely unaffected by the 2 interruptions and only had a statistically significant sustained increase because of a time trend change in the period after the third wave. Antenatal clinic visits were largely unaffected by the 2 pandemic waves, but the drop in postnatal care visits was statistically significant.

We observed a 57% increase in the maternal death rate at the first interruption (wave 2) and a >2-fold increase in maternal deaths at the second interruption (wave 3). After each of those increases, we noted a substantial, although statistically nonsignificant, waning of the death rate over the course of each wave. The SARS-CoV-2 Beta variant of concern was in circulation during the second wave in Malawi and the Delta variant was circulating during the third wave (*12*). In the United Kingdom, the Beta and Delta variants were associated with maternal deaths in pregnant and recently pregnant women (*13*), findings which are comparable to ours. Our results are also comparable to findings on a multinational cohort of pregnant women that reported that COVID-19 resulted in increased maternal deaths and exacerbated effects on women in low- and middle-income countries (*14*). Observed increased mortality rates at the beginning of the second and third pandemic waves could be the result of direct effects of COVID-19, such as respiratory failure, coupled with observed lack of invasive ventilation in healthcare facilities in Malawi. Another study in Malawi reported that shortness of breath had a statistically significant association with maternal death during the second and third COVID-19 waves (*15*).

Although the drop in neonatal death rate at the onset of both the second and third COVID-19 waves was not statistically significant, the sustained drop after the third COVID-19 wave was significant. Neonatal mortality rate remained below anticipated numbers compared with the counterfactual model of no association. A systematic review of evidence regarding maternal, fetal, and neonatal death associated with COVID-19 showed that direct COVID-19 infection of neonates was not associated with statistically significant mortality rates (*16*). Similarly, our data show that COVID-19 does not seem to have caused overall adverse effects on the outcomes of neonates in Malawi, suggesting that care during labor and the neonatal period was relatively available, despite the challenges to healthcare facilities caused by COVID-19.

Across the 2 COVID-19 waves, we observed fewer stillbirths than anticipated under the counterfactual model. We noted an increase in stillbirths at the onset of the third wave, but that was not sustained. Other studies have found no relationship between SARS-CoV-2 infection and stillbirths, which also appeared to be true for Malawi. In Nepal, the lockdown, and not necessarily COVID-19 infection, was associated with an increase in stillbirths (*4*). Different studies have also reported increased preterm birth outcomes after SARS-CoV-2 infection, but not increases in stillbirths (*17*,*18*).

Despite the COVID-19 waves, antenatal clinic visits remained largely unaffected in Malawi. The country implemented a multisectoral response policy to respond the COVID-19 pandemic, which included risk communication, community engagement, and ensuring that critical services such as antenatal clinics remained robust (*19*).

Although antenatal clinic visits were largely unaffected, postnatal care clinical visits showed a sharp decline at the start of the second COVID-19 wave. About 6 weeks after discharge from facilities, many women did not return for care as recommended, which might be attributed to fear of SARS-CoV-2 infection, as seen among other patients in facilities (*20*). The reluctance to return for care might also have been linked to instituted facility-level policy changes that did not actively encourage women to return. Researchers in the United Kingdom called for vigilance to avoid disruptions and improve maternal postnatal healthcare amidst COVID-19 (*21*). However, evidence suggests that a substantial number of postnatal women became fearful of COVID-19 and more concerned about their wellbeing and that of their babies if exposed to SARS-CoV-2 in crowded environments (*20*). This fear likely explains why, in Malawi, postnatal care visits dropped substantially throughout our study period. Further qualitative research could examine the drop-in postnatal care visits and needed health education. The drop-in postnatal care visits also might have affected women’s opportunities for critical postnatal interventions, such as contraception, which could have lasting effects that will require further evaluation and mitigation.

The first limitation of our study is that we attributed the baseline to a period when Malawi had already experienced the first COVID-19 wave. MATSurvey was not optimally functional before COVID-19 emerged, which is why we chose the period after the first wave as baseline in the interrupted regression analysis. Second, we chose to focus on 2 key interruptions, the second and third COVID-19 waves, because those presented the most critical scenarios in the country. Third, all the outcomes investigated in this study are characterized by substantial amounts of variation not explained by our models. Although some random variation existed, other drivers for variation in maternal and neonatal death, stillbirths, and antenatal and postnatal visits likely were not included in our data and hence could not accounted for in our statistical models. A limitation of the dataset is that MATSurvey does not include data on possible confounders; hence, we could not adjust for those data in the ITS regression models. Fourth, our data might have risked overfitting, which we mitigated by choosing the 2 interruption time points in the regression models a priori, and we did not change those time points from outcome to outcome except for postnatal care visits, for which we had to account for the 6-week postnatal lag. Finally, our prediction 95% CIs for the hypothetical models are large and widen as the predictions stretched beyond January 1, 2021, the first interruption timepoint. Although not unexpected, because we were predicting beyond the data range used for fitting the model, the widening predictions mean considerable uncertainty exists regarding our estimates of differences between counterfactual model predictions and ITS regression fits. Those differences are reflected in the reported CIs but do not mean that interpretation of those findings needs to be nuanced, which affects all outcomes, but it is particularly acute for the maternal death outcome.

In conclusion, we used an ITS model to investigate the effects of the 2 most critical COVID-19 waves on maternal and neonatal health indicators in Malawi, which might also apply to other countries in sub-Saharan Africa. Further studies are needed to comprehend the burden of COVID-19 on maternal, neonatal, and child health outcomes. Nonetheless, our findings demonstrate the need for strengthening maternal, neonatal, and child healthcare systems to prepare for future pandemics, particularly in resource-constrained settings.
